# Fish consumption and mortality in the European Prospective Investigation into Cancer and Nutrition cohort

**DOI:** 10.1007/s10654-014-9966-4

**Published:** 2014-11-07

**Authors:** Dagrun Engeset, Tonje Braaten, Birgit Teucher, Tilman Kühn, H. B. Bueno-de-Mesquita, Max Leenders, Antonio Agudo, Manuela M. Bergmann, Elisavet Valanou, Androniki Naska, Antonia Trichopoulou, Timothy J. Key, Francesca L. Crowe, Kim Overvad, Emily Sonestedt, Amalia Mattiello, Petra H. Peeters, Maria Wennberg, Jan Håkan Jansson, Marie-Christine Boutron-Ruault, Laure Dossus, Laureen Dartois, Kuanrong Li, Aurelio Barricarte, Heather Ward, Elio Riboli, Claudia Agnoli, José María Huerta, María-José Sánchez, Rosario Tumino, Jone M. Altzibar, Paolo Vineis, Giovanna Masala, Pietro Ferrari, David C. Muller, Mattias Johansson, M. Luisa Redondo, Anne Tjønneland, Anja Olsen, Karina Standahl Olsen, Magritt Brustad, Guri Skeie, Eiliv Lund

**Affiliations:** 1 Department of Community Medicine, UiT-The Arctic University of Norway, Tromsö, Norway; 2 Department of Translational Pulmonology, Translational Lung Research Center Heidelberg (TLRC), University of Heidelberg, Heidelberg, Germany; 3 German Cancer Research Center (DKFZ), Heidelberg, Germany; 4 National Institute for Public Health and the Environment, Bilthoven, The Netherlands; 5 Department of Gastroenterology and Hepatology, University Medical Centre, Utrecht, The Netherlands; 6 School of Public Health, Imperial College London, London, UK; 7 Institute for Risk Assessment Sciences, Utrecht University, Utrecht, The Netherlands; 8 Unit of Nutrition, Environment and Cancer, IDIBELL, Catalan Institute of Oncology-ICO, Barcelona, Spain; 9 German Institute of Human Nutrition Potsdam-Rehbrücke, Potsdam, Germany; 10 Hellenic Health Foundation, Athens, Greece; 11 Department of Hygiene, Epidemiology and Medical Statistics, School of Medicine, University of Athens, Athens, Greece; 12 WHO Collaborating Center for Food and Nutrition Policies, Department of Hygiene, Epidemiology and Medical Statistics, University of Athens Medical School, Athens, Greece; 13 Cancer Epidemiology Unit, Nuffield Department of Population Health, Oxford, UK; 14 Section for Epidemiology, Department of Public Health, Aarhus University, Aarhus, Denmark; 15 Department of Clinical Sciences in Malmö, Lund University, Lund, Sweden; 16 Dipartimento Di Medicina Clinica E Chirurgia Federico II University, Naples, Italy; 17 Department of Epidemiology, Julius Center for Health Sciences and Primary Care, University Medical Center, Utrecht, The Netherlands; 18 Department of Epidemiology and Biostatistics, Faculty of Medicine, School of Public Health, Imperial College London, London, UK; 19 Department of Public Health and Clinical Medicine, Nutritional Research, Umeå University, Umeå, Sweden; 20 Department of Public Health and Clinical Medicine, Research Unit Skellefteå, Umeå University, Umeå, Sweden; 21 Inserm, Centre for research in Epidemiology and Population Health (CESP), U1018, Nutrition, Hormones and Women’s Health Team, Villejuif, France; 22 Univ Paris Sud, UMRS 1018, Villejuif, France; 23 IGR, 94805 Villejuif, France; 24 Navarre Public Health Institute, Pamplona, Spain; 25 Consortium for Biomedical Research in Epidemiology and Public Health (CIBER Epidemiología y Salud Pública-CIBERESP), Madrid, Spain; 26 Department of Epidemiology and Biostatistics, School of Public Health, Imperial College London, London, UK; 27 Epidemiology and Prevention Unit, Fondazione IRCCS Istituto Nazionale dei Tumori, Milan, Italy; 28 Department of Epidemiology, Murcia Regional Health Council, Murcia, Spain; 29 Escuela Andaluza de Salud Pública, Instituto de Investigación Biosanitaria de Granada (Granada.ibs), Granada, Spain; 30 Cancer Registry and Histopathology Unit, “Civic-. M. P. Arezzo” Hospital, ASP Ragusa, Ragusa, Italy; 31 Public Health Department of Gipuzkoa, San Sebastian, Spain; 32 HuGeF Foundation, Turin, Italy; 33 Molecular and Nutritional Epidemiology Unit, Cancer Research and Prevention Institute – ISPO, Florence, Italy; 34 International Agency for Research on Cancer, Lyon, France; 35 Public Health Directorate, Asturias, Spain; 36 Danish Cancer Society Research Center, Copenhagen, Denmark

**Keywords:** Mortality, Fish consumption, Cohort, Lean fish, Fatty fish, Multi-centre study

## Abstract

Fish is a source of important nutrients and may play a role in preventing heart diseases and other health outcomes. However, studies of overall mortality and cause-specific mortality related to fish consumption are inconclusive. We examined the rate of overall mortality, as well as mortality from ischaemic heart disease and cancer in relation to the intake of total fish, lean fish, and fatty fish in a large prospective cohort including ten European countries. More than 500,000 men and women completed a dietary questionnaire in 1992–1999 and were followed up for mortality until the end of 2010. 32,587 persons were reported dead since enrolment. Hazard ratios and their 99 % confidence interval were estimated using Cox proportional hazard regression models. Fish consumption was examined using quintiles based on reported consumption, using moderate fish consumption (third quintile) as reference, and as continuous variables, using increments of 10 g/day. All analyses were adjusted for possible confounders. No association was seen for fish consumption and overall or cause-specific mortality for both the categorical and the continuous analyses, but there seemed to be a U-shaped trend (*p* < 0.000) with fatty fish consumption and total mortality and with total fish consumption and cancer mortality (*p* = 0.046).

## Introduction

Fish is a source of many important nutrients, such as high quality proteins, vitamins A, B, D and E, minerals like iron, zinc, selenium and iodine, and the marine omega-3 fatty acids (eicosapentaenoic, EPA, and docosahexaenoic, DHA, acids) [3], and according to a report from the joint Food and Agriculture Organization/World Health Organization (FAO/WHO) expert consultation [49], there is convincing evidence that fish consumption lowers the risk of death from coronary heart disease. However, research on fish consumption and cancer risk is not as encouraging; a report from the World Cancer Research Fund/American Institute for Cancer Research (WCRF/AICR) only found limited-suggestive evidence for decreased risk of colorectal cancer with fish consumption [48].

Studies on all-cause mortality and fish consumption are also inconclusive. In a Danish cohort study on both men and women they found that men who were eating fish once a month or less had lower all-cause mortality compared with those eating fish once a week, and that all-cause mortality seemed to increase with increasing frequency of fish intake [28]. A similar study from the USA found a reduced risk of all-cause mortality in white men consuming fish once a week compared to never consumption, but not in black men and women [16]. In a case–control study in Hong Kong Chinese, higher consumption of one to three times a week was associated with lower mortality compared with the lowest fish consumption of less than or equal to three times a month [44].

Studies on fish consumption and cancer diseases and mortality are inconclusive, thus it may not be the fish itself that provide benefits but rather the fact that fish replaces other less healthy foods previously shown to increase the risk, e.g. red meat [5].

Fish is unfortunately also a source of environmental pollutants, e.g. methyl mercury (MeHg) and persistent organic pollutants, like PCB and DDT among others [49]. This complicates the risk–benefit assessment for fish consumption. The benefits from eating fish versus not eating fish may be outweighed by the harmful effects of environmental pollutants. The Joint FAO/WHO Expert Committee have compared the risks and benefits of eating fish, and although they found convincing evidence that high dioxin exposure increases the risk of cancer, their conclusion was that potential cancer risks of dioxins are well below established coronary heart disease benefits [49]. They recommend an intake of 1–2 portions of fatty fish a week. Based on the current knowledge of risk and benefits from eating fish, many countries have recommendations of 2–3 portions (100–150 g/portion) of a variety of fish a week [15, 38–40, 42].

In studies where fish consumption is used as an exposure variable, observed benefits are often attributed to the presence of the fatty acids, despite the fact that the concentration of fatty acids in fish varies considerably depending on the species, season, etc. In lean (white) fish, meat contains only small amounts of fat, because most of the fat is deposited in the guts (liver and row), whereas in fatty (dark meat) fish the fat is found intramuscularly and will provide a higher amount of fatty acids [3]. It is therefore important to distinguish between lean and fatty fish in the analyses.

In the European Prospective Investigation into Cancer and Nutrition (EPIC) study, we have previously found no association between fish consumption and breast cancer [9], lung cancer [21], and pancreatic cancer [31], and a slight preventive effect of fish consumption on colon cancer [26] and hepatocellular carcinoma [11]. However, only the studies on breast cancer and hepatocellular carcinoma were studying sub-types of fish, and none of them have looked specifically at cancer mortality.

In the present study we examined overall mortality and death from ischaemic heart disease and cancer in relation to both total fish consumption and lean and fatty fish consumption separated, in the EPIC cohort.

## Materials and methods

The EPIC study has been presented in detail earlier [30, 37], thus, only a short overview is given here. The EPIC cohort consists of participants from 23 centres in ten countries; Spain, Greece, France, Italy, Germany, the Netherlands (NL), the United Kingdom (UK), Denmark, Sweden, and Norway.

## Study participants

The EPIC cohort included 509,308 women and men, aged mostly 35–70 years at enrolment (1992–1998/1999). The study participants were recruited from the general population and within defined areas in each country with some exceptions; participants were female members of a health insurance scheme for state school employees in France, women attending breast cancer screening in Utrecht (NL) and Florence (Italy), and units of the Italian and Spanish cohorts included members of local blood donor associations. In Oxford (UK), a part of the cohort includes persons not consuming meat (further referred to as health-conscious). In Norway, France, Naples (Italy), and Utrecht only women were recruited [30].

Eligible participants gave written informed consent and completed questionnaires on their diet, lifestyle, and medical history. Approval for this study was obtained from the ethical review boards of the International Agency for Research on Cancer and from all local institutions where subjects had been recruited for the EPIC study.

Individuals in the top and bottom 1 % of the ratio of energy intake to estimated energy requirement (calculated from age, sex and bodyweight) were excluded from the analyses to reduce the effect of implausible extreme values (n = 1,033), in addition 6,627 persons with missing dietary data and 21,113 persons with missing info on non-dietary covariates were excluded. The number of subjects included in the analysis of total fish was 480,535. Due to missing information on lean and fatty fish in the cohorts from Naples, Heidelberg (Germany), Potsdam (Germany) and Umeå (Sweden), we had to exclude all 79,324 individuals from these centres, leaving 401,211 subjects for the separate analysis on lean and fatty fish.

## Diet and lifestyle questionnaire

Dietary data were obtained using different validated dietary history or food-frequency questionnaires (FFQ), tailored to each country in order to capture local dietary habits and provide high compliance. To calibrate dietary data collected by different instruments, a single 24-h computerized dietary recall (24-HDR) was performed in a random sample (8 %) of the EPIC cohort (36,900 individuals) [36, 37]. The aim of the calibration study was to adjust for systematic differences in reporting of dietary intakes across countries due to different populations and different instruments [14]. Fish intake has been evaluated in the EPIC cohort, and strong correlations were found between fish intake and plasma phospholipid fatty acids [33].

We examined total fish consumption (lean and fatty fish), lean fish consumption, and fatty fish consumption. Products of lean fish (e.g. fish cakes, fish stew, and similar) were not included in the analyses. Fatty fish consumption included canned fish products. Fish containing <4 % fat was classified as lean (e.g. cod, haddock, plaice), whereas fish containing 4 % fat or more was classified as fatty (e.g. salmon, trout, herring, mackerel).

Questions about fish consumption in the questionnaires varied considerably between centres and countries, from simple questions on whether participants ate fish and the frequency and portion size to more detailed information on type of fish eaten, cooking methods and seasonal variability.

Lifestyle questionnaires included questions about education, socio-economic status, occupation, history of previous illness, physical activity, anthropometry, alcohol consumption and smoking status.

## End points

Follow-up was based on cancer registries, boards of health, and death indices in Denmark, Italy (except Naples), the Netherlands, Spain, Sweden, Norway, and the United Kingdom. In France, Germany, Greece, and Naples (Italy) this information was obtained from municipality registries, regional health departments, physicians, and hospitals, and by contacting next-of-kin. The participants were followed from enrolment (1992–1999) until death, emigration or end of the follow-up period (Varese and Naples (Italy) 2006; Florence (Italy) 2007; Granada, Murcia, and San Sebastian (Spain), Malmö (Sweden), and Denmark 2008; Ragusa (Italy), Asturias and Navarra (Spain), Oxford (UK), The Netherlands, Greece, Umeå (Sweden), and Norway 2009; Turin (Italy), Cambridge (UK), Germany, and France 2010).

Causes of death reported in death certificates were recorded according to the 10th edition of the International Statistical Classification of Diseases, Injuries and Causes of Death (ICD10), with ischaemic heart death defined as I20–25, and cancer death of all causes as C00–97 (malignant neoplasms).

## Statistical methods

Due to the large sample size and statistical power of the study we decided to use a more stringent confidence interval, thus, hazard ratios (HR) and their 99 % confidence interval (CI) were estimated using Cox proportional hazard regression models. Attained age was used as the primary time variable in the Cox regression models. The analyses were stratified by centre and age at enrolment in 1 year intervals to control for effects related to different follow-up procedures and questionnaire design. France was included as a single centre, as dietary assessment and follow-up procedures were the same throughout the country, as was the case for Norway. The UK Oxford centre was divided into two, one for the general population and one for the health conscious participants.

Fish consumption, as reported in the questionnaires, was divided into EPIC-wide quintiles to ensure than comparisons were made over the variability in intake of the entire EPIC cohort. Models with fish consumption as a continuous variable, using an increment of 10 g/day, were also examined. To account for the variability of fish consumption within the different countries fish consumption was also divided into country-wide quintiles. Sex-specific analyses as well as combined analyses of total fish were performed. To investigate which food items higher fish consumption replaced we performed sex and country specific analysis where we looked at consumption of red meat, processed meat, and white meat in quintiles of total fish intake, adjusted for total energy and mutually for each other.

To correct for centre-specific bias and regression dilution within each centre stratum, the 24-HDR values for participants of the calibration study were regressed on their main study dietary questionnaire values, providing regression coefficients for fish consumption.

Age at recruitment, weight, BMI, and season in which the FFQ data were collected were included as covariates in the calibration model. In addition, centre was included in the calibration model as a main effect to ensure correction for between-centre measurement errors. Estimation of regression coefficients was weighted for season and day (weekday/weekend) of the 24-HDR measurements [16].

The regression intercepts and slopes that were obtained from the calibration study were then applied to the main study questionnaire data to obtain individual predicted values of dietary exposure for all participants. Cox regression models were conducted using the predicted values for each individual. An indicator variable (non-consumer/consumer) was included in the disease model. The same covariates were included in disease models using calibrated values as for the non-calibrated model. The middle quintile was used as reference since they are considered to be more “normal consumers” than the non/very low-consumers, who are often different from the rest in many ways (e.g. vegetarians, allergies, etc.). The middle quintile is also closer to the recommended intake of fish [15, 38–40, 42]. The standard error of the de-attenuated coefficient was calculated with bootstrap sampling (ten samples) in the calibration models to take into account the uncertainty related to measurement error correction.

We explored fish intake using different models for energy adjustment (substitution model, and residual method, model 1 (disease = b_1_ Nutrient residual) and model 2 (disease = b_1_ Nutrient residual + b_2_ Calories). Nutrient residual is the residual from the regression of a specific nutrient (b_1_) on calories and b_2_ Calories represent calories provided by the specific nutrient) [47], but this did not appreciably change the observed associations and we decided to use the substitution model in our final analysis. In the substitution model, the energy percentage (E %) of fat, and protein and carbohydrate were included as variables. By including these macronutrients, it is possible to estimate relevant contrasts between macronutrient effects. The interpretation of these two estimated parameters is the effect of increasing the intake of one, while keeping the other constant, i.e., at the expense of the nutrient not included as a variable in the model [10]. Fatty fish and lean fish were mutually adjusted for. In addition, the results were adjusted by including the following covariates in the models: estimated energy intake divided into energy from fat (expressed as 100 g/day), and energy from carbohydrates and proteins (expressed as 100 g/day), total dietary fibres, red meat, processed meat, vegetables, fruit (all continuous in g/day), and categories for alcohol intake (abstainers, <15, 15–30, ≧30 g/day), body mass index (BMI) (<18.5, 18.5–24.9, 25–29.9, ≧30), physical activity in leisure time (inactive, moderate, intense, unknown = 1.1 %), smoking status (never smoker, former—quit ≧10 years ago, former—quit <10 years ago, former—quit unknown, current <15 cigarettes/day, current 15–24 cigarettes/day, current ≧25 cigarettes/day, current—number of cigarettes unknown, smoking status unknown = 1.7 %), education (none, primary school, technical/professional school, secondary school, university degree, not specified = 2.4 %).

Test for heterogeneity across countries was performed with Likelihood ratio test, and Wald test was used to test for heterogeneity between men and women.

To account for the distance between the quintiles, a variable with the median within each quintile was included. The same variable was then included as squared, to test for linear and quadratic (U-shaped) trend.

Restricted cubic spline regression was used to examine non-linearity of the relative risk function for predicted intake data. Log likelihood ratio statistics was used to evaluate whether the fish variables contribute significantly to the model fit, either for the linear or the restricted cubic spline model, and whether the restricted cubic spline model parameters add significantly to the model fit compared to the linear model.

To rule out reverse causation the analyses were repeated excluding participants with <2 years of follow up.

All analyses were performed using SAS version 9.2 (SAS Institute, Cary, North Carolina).

## Results

Out of the total 480,535 included participants, 32,587 persons were reported dead in the EPIC cohort during follow-up. Mean total fish intake for the total cohort was 27 g/day for men, with highest intake in Spanish men (mean of 69 g/day), and 29 g/day for women, with highest intake in Norwegian women (mean of 73 g/day) (Table 1) (calibrated numbers).

**Table 1:** Characteristics of the EPIC cohort, based on FFQ information

Country	Cohort (n)		Deaths (n)		Mean age		Mean total fish intake, g/day (range)	
	Men	Women	Men	Women	Men	Women	Men	Women
France		69,980		3,746		52.9		33.6 (0–295.1)
Italy	14,279	30,525	680	899	50.3	50.7	24.7 (0–228.2)	24.4 (0–254.2)
Spain	15,243	25,067	1,148	752	50.7	48.4	68.7 (0–471.1)	47.0 (0–357.1)
UK	23,001	54,630	3,639	3,996	53.3	47.9	26.9 (0–585.3)	26.3 (0–707.9)
The Netherlands	9,720	28,158	525	1,779	43.3	51.3	5.1 (0–84.9)	4.8 (0–138.3)
Greece	10,942	15,627	1,256	808	52.9	53.4	21.3 (0–573.6)	17.9 (0–189.9)
Germany	22,363	29,620	1,793	966	52.6	49.3	18.8 (0–379.3)	14.6 (0–388.3)
Sweden	21,105	25,049	2,708	1,808	51.9	51.8	15.4 (0–300.3)	13.4 (0–174.6)
Denmark	26,530	29,175	3,235	2,095	56.6	56.8	34.3 (0–316.4)	29.4 (0–306.2)
Norway		29,521		754		48.0		73.2 (0–678.2)
Total cohort	143,183	337,352	14,984	17,603	51.5	51.1	26.9 (0–585.3)	28.5 (0–707.9)

Persons with lowest mean fish intake (lowest quintile) had the lowest mean intake of alcohol, vegetables, fruit, and meat (Table 2). Persons in the lowest quintile of fish intake tended to be younger, have a lower BMI, have a university degree, and for men we saw more never smokers. For women, the highest percentage of current smokers was observed in the first quintile, whereas never smokers were almost equally distributed among the three middle quintiles. A higher fish intake generally corresponded with a higher consumption of fruit and vegetables and meat for both genders, and with a higher physical activity level. Men in the highest quintile tended to have a higher intake of alcohol, and there were more current smokers (Table 2).

**Table 2:** Sex-specific baseline information by EPIC-wide quintiles of total fish consumption in the EPIC study

	Q1	Q2	Q3	Q4	Q5
*Men*					
Age at recruitment (years, mean/median)	47.8/49.4	51.4/51.6	54.1/54.2	54.5/54.7	53.9/53.9
BMI (kg/m^2,^ mean/median)	25.6/25.3	26.4/26.0	26.6/26.3	26.7/26.3	27.3/26.9
Alcohol intake (g/day, mean/median)	15.7/8.5	19.2/11.5	21.8/14.3	23.3/16.1	26.1/18.3
Vegetable intake (g/day, mean/median)	145.6/113.0	175.7/124.9	194.9/153.4	211.6/173.1	248.2/214.4
Fruit intake (g/day, mean/median)	166.7/124.0	198.1/145.5	199.6/154.3	210.6/163.2	266.1/216.9
Total meat intake(g/day, mean/median)	83.6/75.2	92.7/82.8	99.0/91.7	105.3/98.8	107.9/99.8
Total energy intake (kcal, mean/median)	2,274.5/2,202.3	2,319.2/2,246.5	2,369.5/2,298.6	2,478.5/2,415.6	2,632.9/2,572.3
Never smoker (%)	39.8	33.8	31.1	29.8	29.2
Former smoker (%)	33.3	35.0	39.6	39.3	37.2
Current smoker (%)	26.3	29.9	28.2	30.1	33.1
University degree (%)	28.9	25.8	27.6	27.7	23.8
Physical activity, intense (%)	30.2	31.5	32.5	34.4	36.0
*Women*					
Age at recruitment (years, mean/median)	47.8/49.7	51.6/51.7	52.1/52.0	52.1/51.9	51.2/50.8
BMI (kg/m^2^,mean/median)	24.5/23.7	25.2/24.3	24.9/24.1	25.0/24.1	25.1/24.3
Alcohol intake (g/day, mean/median)	7.3/3.0	8.3/3.6	9.4/5.2	9.4/5.2	8.0/3.6
Vegetable intake (g/day, mean/median)	181.7/142.3	201.8/161.5	225.0/195.0	241.5/216.2	251.8/223.3
Fruit intake (g/day, mean/median)	221.1/184.1	238.6/205.5	245.8/212.7	255.8/223.9	261.9/225.1
Total meat intake(g/day, mean/median)	53.7/46.9	65.3/59.6	68.5/64.0	71.3/66.4	67.4/62.1
Total energy intake (kcal, mean)	1,794.3/1,745.0	1,868.3/1,813.5	1,952.5/1,897.1	2,037.3/1,982.6	2,025.4/1,954.0
Never smoker (%)	52.9	57.7	57.9	57.7	52.0
Former smoker (%)	25.0	20.9	22.2	22.4	24.2
Current smoker (%)	21.5	19.8	17.8	17.5	20.5
University degree (%)	26.0	22.4	24.1	23.5	21.7
Physical activity, intense (%)	30.0	31.8	33.0	34.7	35.7

When looking at the different types of meat to find what higher fish consumption substituted for no consistent relationship was seen for red meat. A positive trend was seen in some countries, negative in others, but the trend was non-linear in most countries. Intake of processed meat decreased with increasing intake of fish, though not entirely unambiguous. The clearest and most consistent relationship was seen for white meat which increased with increasing fish intake in all countries (data not shown).

The correlation between the intake of fatty and lean fish was moderate (Pearsons r = 0.37).

When testing the predicted data for non-linearity by restricted cubic spline regression, the likelihood ratio test revealed that the spline model did not improve the linear model fits.

Excluding participants with <2 years of follow up did not change the estimates notably for any of the analyses.

### Total death, ischaemic heart disease death, and cancer death

The statistical significant associations found in the non-calibrated analyses disappeared in the calibrated analyses and bootstrap analyses. No associations were seen for consumption of total fish, lean, or fatty fish and either total mortality or cause-specific mortality among men; broadly similar results were obtained for women (Tables 3, 4, 5). No associations were seen in the continuous analyses of fish consumption (Table 3).

**Table 3:** Risk of overall mortality in relation to fish consumption in quintiles (third quintile as reference), and as a continuous variable with 10 g increment

	Q1	Q2	Q3	Q4	Q5	Continuous, 10 g increment
Male						
*Total fish consumption (g/d)*	1.9	10.8	21.1	34.2	76.2	
*N cases*	2,427	2,769	3,334	3,407	3,047	14,984
HR crude^a^	0.98 (0.77–1.26)	0.98 (0.78–1.22)	Ref	1.11 (0.91–1.35)	1.08 (0.86–1.35)	1.01 (1.00–1.02)
HR uncalibrated	1.04 (0.97–1.12)	1.05 (0.97–1.12)	Ref	1.05 (0.98–1.11)	**1.16 (1.08–1.24)**	**1.01 (1.01–1.02)**
HR calibrated	0.96 (0.85–1.08)	0.95 (0.84–1.07)	Ref	1.02 (0.91–1.16)	1.04 (0.93–1.18)	1.01 (1.00–1.03)
*Lean fish consumption (g/d)*	0.1	4.0	11.8	20.6	50.4	
*N cases*	2,217	1,782	2,283	3,378	2,764	12,424
HR crude^a^	**1.14 (1.04–1.25)**	**1.14 (1.04–1.24)**	Ref	1.08 (1.00–1.16)	**1.16 (1.07–1.25)**	1.01 (1.00–1.02)
HR uncalibrated	**1.14 (1.04–1.25)**	**1.11 (1.02–1.22)**	Ref	**1.10 (1.02–1.18)**	**1.19 (1.10–1.28)**	1.02 (1.00–1.03)
HR calibrated	0.96 (0.84–1.10)	0.98 (0.85–1.12)	Ref	1.01 (0.88–1.16)	1.01 (0.89–1.16)	1.02 (0.98–1.05)
*Fatty fish consumption (g/d)*	0.1	2.9	7.8	14.1	35.6	
*N cases*	2,648	2,150	2,666	2,409	2,551	12,424
HR crude^a^	1.00 (0.93–1.08)	1.00 (0.92–1.09)	Ref	0.91 (0.84–0.98)	0.99 (0.92–1.07)	1.00 (0.98–1.01)
HR uncalibrated	0.98 (0.91–1.06)	0.98 (0.90–1.06)	Ref	0.93 (0.86–1.00)	1.02 (0.94–1.10)	1.00 (0.99–1.02)
HR calibrated	1.10 (0.97–1.25)	1.07 (0.94–1.22)	Ref	1.08 (0.95–1.23)	1.06 (0.93–1.21)	1.00 (0.97–1.03)
Female						
*Total fish consumption (g/d)*	1.9	10.8	21.1	34.2	76.2	
*N cases*	3,403	3,383	3,728	3,690	3,399	17,603
HR crude^a^	1.04 (0.68–1.58)	0.99 (0.75–1.31)	Ref	0.96 (0.74–1.25)	0.97 (0.65–1.45)	1.00 (0.99–1.01)
HR uncalibrated	1.07 (1.00–1.15)	1.03 (0.96–1.10)	Ref	0.99 (0.93–1.05)	1.03 (0.96–1.10)	1.00 (0.99–1.01)
HR calibrated	1.05 (0.94–1.18)	0.99 (0.88–1.10)	Ref	1.02 (0.91–1.14)	1.06 (0.95–1.18)	1.01 (0.98–1.03)
*Lean fish consumption (g/d)*	0.1	4.1	11.5	20.5	52.0	
*N cases*	3,525	3,131	2,979	3,556	2,910	16,101
HR crude^a^	**1.12 (1.05–1.20)**	1.05 (0.98–1.13)	Ref	0.99 (0.93–1.06)	1.04 (0.96–1.11)	1.00 (0.99–1.01)
HR uncalibrated	**1.10 (1.03–1.18)**	1.05 (0.98–1.13)	Ref	1.00 (0.94–1.07)	1.04 (0.97–1.12)	1.00 (0.99–1.01)
HR calibrated	1.05 (0.94–1.16)	0.99 (0.89–1.10)	Ref	1.01 (0.91–1.12)	1.01 (0.91–1.12)	1.00 (0.97–1.03)
*Fatty fish consumption (g/d)*	0.2	2.9	7.7	14.1	33.9	
*N cases*	3,602	3,245	3,376	3,101	2,777	16,101
HR crude^a^	**1.11 (1.04–1.19)**	1.07 (1.00–1.14)	Ref	0.97 (0.91–1.03)	1.01 (0.94–1.08)	1.00 (0.98–1.01)
HR uncalibrated	**1.09 (1.02–1.17)**	1.06 (0.99–1.13)	Ref	0.98 (0.92–1.05)	1.03 (0.96–1.10)	1.00 (0.99–1.02)
HR calibrated	1.03 (0.92–1.15)	1.01 (0.90–1.13)	Ref	0.93 (0.83–1.04)	1.09 (0.97–1.22)	1.01 (0.97–1.06)

Data for males and females are presented separately, with 99 % confidence intervals, statistically significant results in bold

Adjusted for energy from fat, energy from carbohydrates and proteins, dietary fibres, red meat, processed meat, vegetables, fruit, alcohol intake, body mass index, physical activity, smoking, education. Lean and fatty fish were mutually adjusted for

^a^Stratified on age, unadjusted, uncalibrated

**Table 4:** Risk of mortality from ischaemic heart disease in relation to fish consumption in quintiles (third quintile as reference), and as a continuous variable with 10 g increment

	Q1	Q2	Q3	Q4	Q5	Continuous, 10 g increment
Male						
*Total fish consumption (g/d)*						
*N cases*	448	524	519	472	252	2,215
HR crude^a^	1.05 (0.87–1.26)	1.04 (0.87–1.24)	Ref	1.01 (0.86–1.19)	1.15 (0.96–1.56)	1.01 (0.99–1.03)
HR uncalibrated	1.03 (0.85–1.23)	1.01 (0.85–1.23)	Ref	1.03 (0.88–1.21)	**1.23 (1.03–1.47)**	1.02 (1.00–1.04)
HR calibrated	0.99 (0.75–1.30)	0.98 (0.74–1.30)	Ref	1.08 (0.82–1.43)	1.09 (0.83–1.44)	1.02 (0.98–1.06)
*Lean fish consumption (g/d)*						
*N cases*	384	244	336	536	444	1,944
HR crude^a^	1.23 (0.98–1.54)	1.08 (0.86–1.37)	Ref	1.15 (0.95–1.39)	**1.31 (1.07–1.60)**	1.03 (0.99–1.06)
HR uncalibrated	1.26 (1.00–1.57)	1.07 (0.85–1.35)	Ref	1.19 (0.98–1.44)	0.96 (0.79–1.17)	1.03 (1.00–1.07)
HR calibrated	0.83 (0.59–1.17)	0.94 (0.67–1.32)	Ref	1.00 (0.71–1.40)	0.95 (0.67–1–33)	1.05 (0.99–1.10)
*Fatty fish consumption (g/d)*						
*N cases*	463	360	438	332	351	1,944
HR crude^a^	1.00 (0.84–1.20)	0.92 (0.75–1.13)	Ref	**0.80 (0.66–0.97)**	0.90 (0.74–1.10)	0.97 (0.93–1.01)
HR uncalibrated	0.97 (0.81–1.16)	0.89 (0.73–1.09)	Ref	0.84 (0.70–1.02)	0.92 (0.83–1.12)	0.99 (0.95–1.03)
HR calibrated	1.14 (0.87–1.49)	1.14 (0.87–1.49)	Ref	1.04 (0.79–1.36)	1.05 (0.80–1.38)	0.99 (0.93–1.05)
Female						
*Total fish consumption (g/d)*						
*N cases*	252	199	227	213	159	1,050
HR crude^a^	1.05 (0.80–1.37)	0.91 (0.70–1.19)	Ref	1.00 (0.78–1.28)	0.90 (0.68–1.19)	0.98 (0.95–1.02)
HR uncalibrated	1.07 (0.82–1.41)	0.91 (0.70–1.19)	Ref	1.00 (0.78–1.28)	0.94 (0.71–1.25)	0.99 (0.95–1.03)
HR calibrated	1.20 (0.79–1.81)	1.16 (0.77–1.75)	Ref	1.18 (0.78–1.77)	1.08 (0.71–1.62)	0.96 (0.89–1.05)
*Lean fish consumption (g/d)*						
*N cases*	212	175	179	216	162	944
HR crude^a^	1.06 (0.80–1.42)	0.97 (0.71–1.31)	Ref	0.88 (0.67–1.16)	0.98 (0.72–1.32)	0.97 (0.92–1.03)
HR uncalibrated	1.10 (0.82–1.48)	1.00 (0.74–1.35)	Ref	0.88 (0.67–1.17)	0.98 (0.72–1.32)	0.96 (0.91–1.02)
HR calibrated	0.98 (0.63–1.51)	0.78 (0.67–1.32)	Ref	0.85 (0.55–1.31)	0.80 (0.52–1.23)	0.93 (0.83–1.03)
*Fatty fish consumption (g/d)*						
*N cases*	256	206	187	174	121	944
HR crude^a^	1.18 (0.91–1.53)	1.11 (0.84–1.48)	Ref	1.07 (0.82–1.41)	1.05 (0.76–1.44)	0.99 (0.93–1.06)
HR uncalibrated	1.18 (0.91–1.54)	1.09 (0.82–1.44)	Ref	1.13 (0.86–1.49)	1.14 (0.83–1.57)	1.03 (0.96–1.10)
HR calibrated	0.97 (0.64–1.47)	1.02 (0.68–1.55)	Ref	1.09 (0.72–1.65)	1.00 (0.66–1.51)	1.03 (0.89–1.19)

Data for males and females are presented separately, with 99 % confidence intervals, statistically significant results in bold

Adjusted for energy from fat, energy from carbohydrates and proteins, dietary fibres, red meat, processed meat, vegetables, fruit, alcohol intake, body mass index, physical activity, smoking, education. Lean and fatty fish were mutually adjusted for

^a^Stratified on age, unadjusted, uncalibrated

**Table 5:** Risk of cancer mortality in relation to fish consumption in quintiles (third quintile as reference), and as a continuous variable with 10 g increment

	Q1	Q2	Q3	Q4	Q5	Continuous, 10 g increment
Male						
*Total fish consumption(g/d)*						
*N cases*	747	711	939	1,012	1,043	4,452
HR crude^a^	1.06 (0.92–1.22)	1.01 (0.88–1.15)	Ref	0.99 (0.88–1.11)	1.06 (0.94–1.20)	1.00 (0.99–1.02)
HR uncalibrated	1.06 (0.92–1.22)	0.98 (0.86–1.13)	Ref	0.99 (0.88–1.12)	1.08 (0.95–1.23)	1.01 (0.99–1.02)
HR calibrated	1.02 (0.82–1.27)	1.01 (0.81–1.26)	Ref	1.03 (0.83–1.28)	1.08 (0.87–1.34)	1.01 (0.99–1.04)
*Lean fish consumption (g/d)*						
*N cases*	711	588	697	1,009	900	3,905
HR crude^a^	1.11 (0.95–1.30)	1.14 (0.97–1.33)	Ref	1.08 (0.94–1.23)	1.11 (0.96–1.27)	1.01 (0.99–1.03)
HR uncalibrated	1.10 (0.94–1.30)	1.12 (0.96–1.31)	Ref	1.09 (0.95–1.24)	1.12 (0.97–1.28)	1.01 (0.99–1.03)
HR calibrated	1.02 (0.83–1.25)	1.01 (0.82–1.23)	Ref	1.08 (0.88–1.33)	1.04 (0.85–1.28)	1.01 (0.98–1.05)
*Fatty fish consumption (g/d)*						
*N cases*	768	623	770	808	936	3,905
HR crude^a^	1.00 (0.87–1.15)	1.05 (0.90–1.22)	Ref	0.97 (0.85–1.11)	1–02 (0.89–1.16)	1.00 (0.97–1.03)
HR uncalibrated	1.01 (0.88–1.16)	1.03 (0.89–1.20)	Ref	0.99 (0.87–1.13)	1.02 (0.89–1.17)	1.00 (0.97–1.03)
HR calibrated	1.07 (0.88–1.31)	0.96 (0.78–1.17)	Ref	1.15 (0.94–1.40)	1.07 (0.88–1.31)	1.00 (0.96–1.05)
Female						
*Total fish consumption (g/d)*						
*N cases*	1,454	1,319	1,501	1,501	1,496	7,271
HR crude^a^	1.05 (0.94–1.17)	1.01 (0.92–1.12)	Ref	0.95 (0.86–1.04)	0.99 (0.90–1.09)	1.00 (0.99–1.01)
HR uncalibrated	1.04 (0.93–1.17)	1.00 (0.91–1.11)	Ref	0.95 (0.87–1.05)	0.99 (0.90–1.00)	1.00 (0.99–1.01)
HR calibrated	1.05 (0.90–1.23)	0.98 (0.84–1.15)	Ref	0.99 (0.85–1.15)	1.03 (0.88–1.20)	1.00 (0.97–1.04)
*Lean fish consumption (g/d)*						
*N cases*	1,472	1,416	1,297	1,396	1,195	6,776
HR crude^a^	1.00 (0.90–1.12)	1.03 (0.92–1.15)	Ref	0.96 (0.87–1.07)	0.95 (0.85–1.07)	1.00 (0.98–1.02)
HR uncalibrated	0.99 (0.89–1.10)	1.02 (0.91–1.14)	Ref	0.96 (0.87–1.07)	0.95 (0.85–1.07)	1.00 (0.99–1.02)
HR calibrated	1.11 (0.94–1.30)	0.99 (0.84–1.17)	Ref	1.03 (0.87–1.21)	1.02 (0.87–1.20)	1.00 (0.96–1.05)
*Fatty fish consumption (g/d)*						
*N cases*	1,377	1,373	1,470	1,290	1,266	6,776
HR crude^a^	0.96 (0.87–1.07)	0.98 (0.88–1.08)	Ref	**0.89 (0.81–0.98)**	0.93 (0.84–1.03)	1.00 (0.97–1.02)
HR uncalibrated	0.96 (0.86–1.06)	0.97 (0.88–1.08)	Ref	**0.90 (0.82–0.99)**	0.94 (0.85–1.04)	1.00 (0.97–1.02)
HR calibrated	1.01 (0.87–1.17)	1.04 (0.89–1.20)	Ref	0.96 (0.83–1.12)	1.07 (0.93–1.24)	1.01 (0.96–1.07)

Data for males and females are presented separately, with 99 % confidence intervals, statistically significant results in bold

Adjusted for energy from fat, energy from carbohydrates and proteins, dietary fibres, red meat, processed meat, vegetables, fruit, alcohol intake, body mass index, physical activity, smoking, education. Lean and fatty fish were mutually adjusted for

^a^Stratified on age, unadjusted, uncalibrated

In the joint analyses of men and women, there were no association with total mortality, ischaemic heart disease mortality, or cancer mortality for fish intake in the continuous analysis, either for total, lean, or fatty fish. However, there seemed to be a U-shaped (*p* < 0.000) trend with fatty fish consumption in the analyses of total mortality, and a U-shaped (*p* = 0.046) trend with total fish consumption in the analyses of cancer mortality (results not shown).

**Fig. 1:**
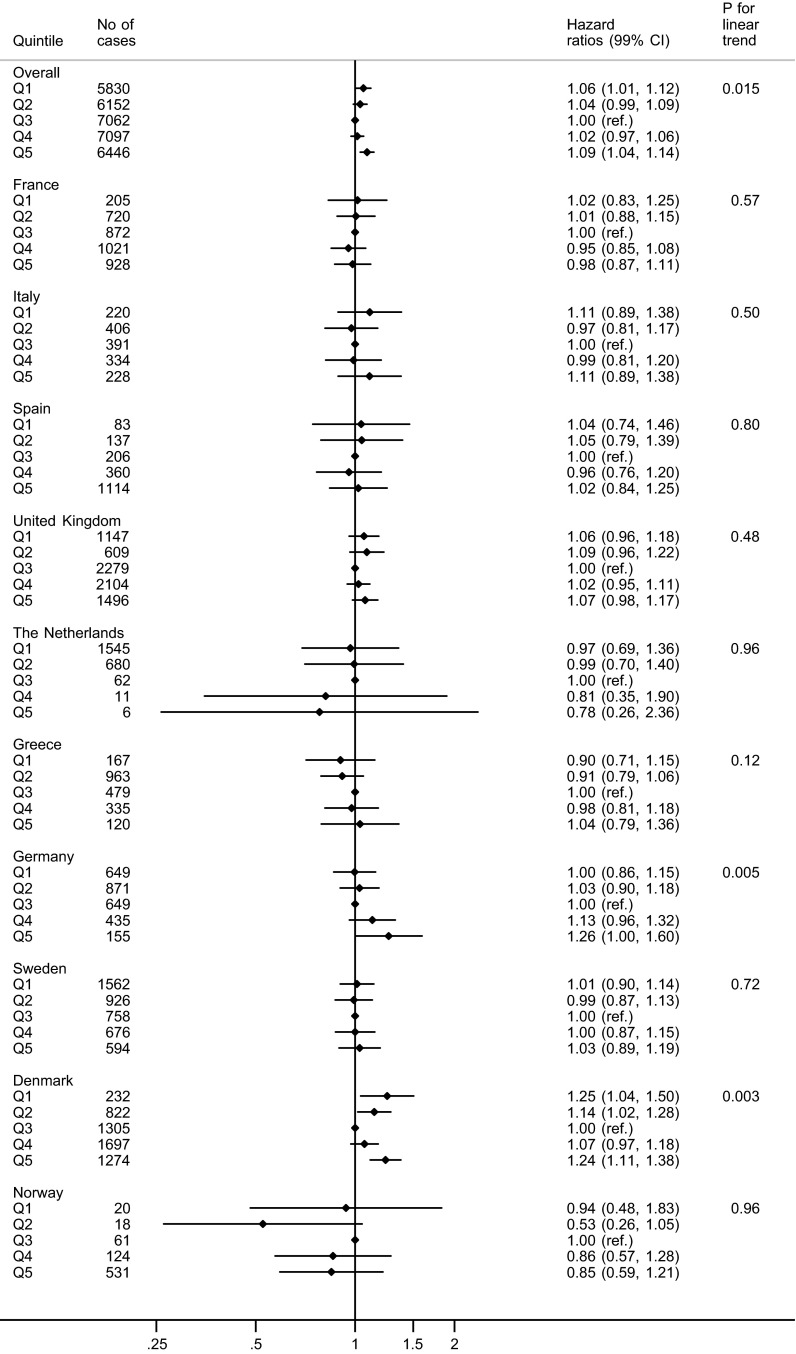
Risk of mortality of all causes by EPIC-wide quintiles of total fish consumption in each country. Uncalibrated data from the EPIC-study

In country-specific analyses we found increased rate of mortality both in the lowest and the highest quintiles compared to the middle quintile for Denmark (Fig. 1). No association was seen for the other countries. *p* value for heterogeneity across countries was 0.03.

The test for heterogeneity between men and women was not significant, except in the analysis of ischaemic heart disease death and lean fish (*p* = 0.01).

**Fig. 2:**
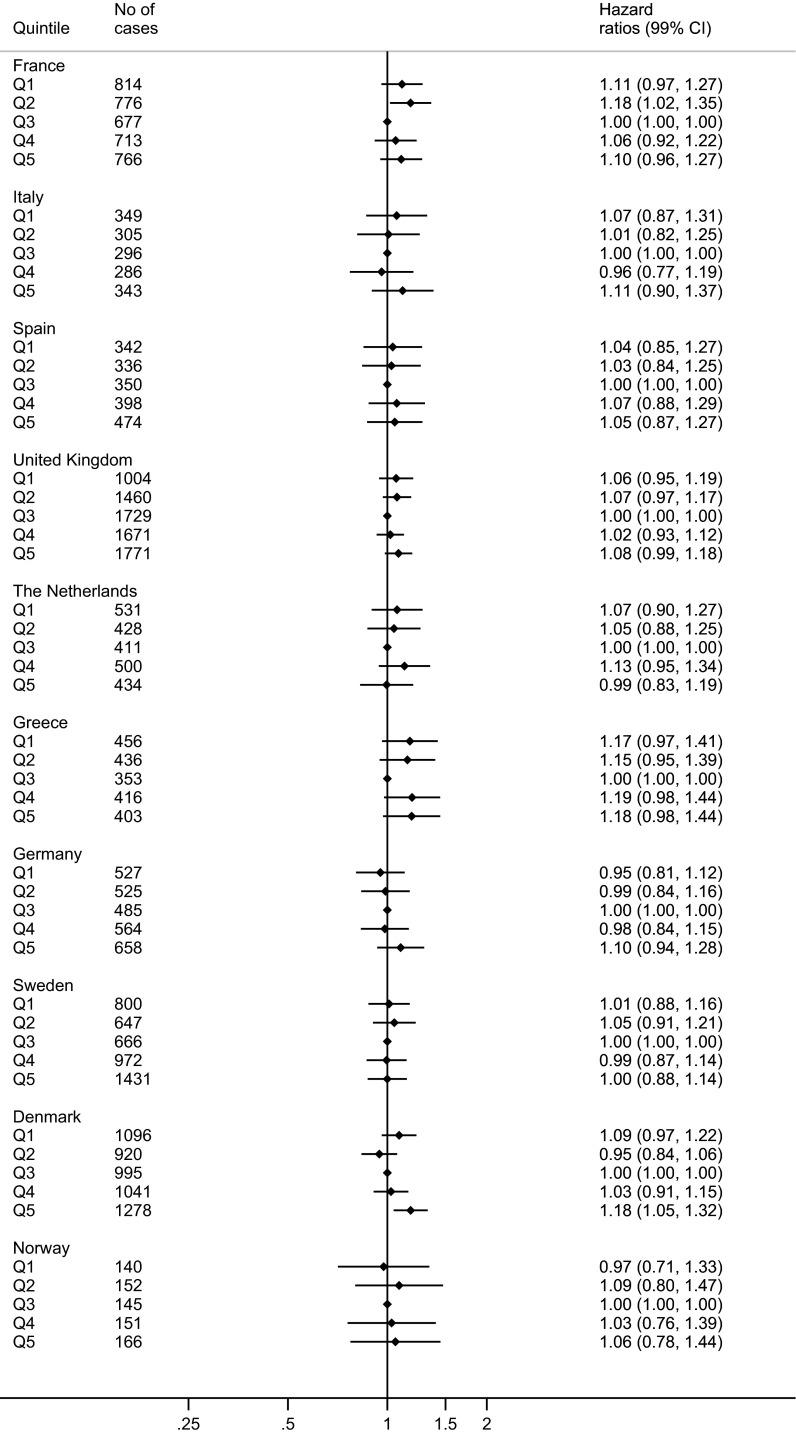
Risk of mortality of all causes by country-wide quintiles of total fish consumption in each country. Uncalibrated data from the EPIC-study

In the country-specific analysis where country-wide quintiles were used instead of EPIC-wide, similar results were seen (Fig. 2). Sex-specific analysis of total fish did not alter the results noteworthy (data not shown).

## Discussion

In this large prospective study of men and women from ten different European countries no association was seen for total, lean or fatty fish consumption and all-cause or cause-specific mortality, neither in the joint analyses nor in the gender-specific analyses of men and women.

For Denmark, a higher rate of all-cause mortality with high as well as with low fish consumption compared to moderate consumption was seen when looking at the different countries separately.

There are several strengths to this study; the prospective design with a large number of participants from different European countries, and the wide range in fish intake. A long follow-up period resulting in a large number of deaths allowed us to distinguish between different causes of death and to examine whether associations were consistent between men and women in the analyses. The single 24-HDR allow for partly correcting for systematic over- and underestimation of dietary intakes [13]. The risk estimates from the calibrated data are considered more reliable than the non-calibrated estimates since calibration may reduce between-centre heterogeneity in the diet-disease relationship caused by differential impact of measurement error across cohorts [12]; however, measurement error may still occur since the errors in the 24-HDR are not completely independent of the errors of the FFQ. Therefore the risk estimates from bootstrapping are considered even more reliable if the sample size is big enough, and bootstrap estimates are preferred when statistical significant values are found [7, 8, 12].

Limitations of this study pertain primarily to our assessment of fish intake. The level of detail concerning fish intake from the different questionnaires varied considerably, and we were not able to consider cooking methods or how the fish was consumed (with sauces, smoked, salted etc.), nor fish oil supplementation, which could also influence the results. Also, reporting bias may differ between populations. Another limitation is the lack of information on contaminants in fish. In addition, reverse causation cannot be completely ruled out, despite the prospective design.

Fish may contain contaminants like MeHg [34], dioxins and polychlorinated biphenyls [35]. Dioxins and polychlorinated biphenyls accumulate in the fat and are therefore more likely to be found in fatty fish. MeHg is found in small amounts in many fish species. The amount differs according to where the fish is caught and species of the fish. It accumulates in the food chain and is therefore more likely to be found in carnivorous fishes, and the amount increases with age and size of the fish [24]. However, according to a review on risk and health benefits of fish consumption and mercury exposure [24], and the joint FAO/WHO report [49], the health risk of not consuming fish outweigh the potential risks from mercury or other contaminants. This was recently supported by two Swedish studies [4, 46], where MeHg, but not reported fish consumption, was associated with cardiovascular health benefits. The explanation suggested for this was that MeHg is a biomarker of fish consumption, independent of reporting bias. Thus, complementing dietary data with biomarkers, when possible, is suggested.

A pooled analysis of eight Asian prospective cohort studies found no association between intake of fish and seafood and risk of all-cause, CVD, or cancer mortality in men, but an inverse association with mortality in women [20]. In contrast to our study, a case–control study in Hong Kong Chinese found a reduced risk of mortality of all causes with higher overall fish intake [45]. Likewise, cohort studies of fishermen and their wives in Finland, Sweden, and Canada respectively, a group of people with high consumption of fish when compared with the general population, also found reduced risk of mortality of all causes [17, 23, 25, 41], whereas similar studies from Denmark and Iceland found increased risks [19, 29]. When looking at the separate countries in our study, we also found an increased risk of mortality in Denmark. The association seems to be U-shaped, with higher risks associated with both high and low consumption compared to moderate consumption. It is difficult to explain why we found this association in Denmark only. The result is based on non-calibrated estimates and may well be a chance finding. However, it may also be due to different way of preparing and consuming fish in Denmark than in the other countries. Fatty fish, especially herring, are popular in Denmark, and the fish is often salted, smoked or pickled [2], hence, the higher risk may be related to the preservation methods of fish. Nitrosamines formed during preservation of food caused cancers in laboratory animals, and are anticipated to be a human carcinogen [1]. Another explanation could be contaminants in fish, or more likely a combination of different factors; ways of preserving and preparing fish, contaminants and lifestyle.

In a large American prospective study, including more than 40,000 male health professionals, Virtanen et al. [43] found that a modest fish consumption (one serving/week) was associated with a lower risk of cardiovascular disease, but not of total cancer or overall major chronic disease, compared to the reference category of <1 serving per month. A Chinese cohort study on fish intake and risk of total and cause specific mortality of 134,296 men and women found no association with death from ischaemic heart disease compared to the reference category with a median intake of 11.1 g fish/day, a reference value closer to our with a mean fish intake of approximately 21 g/day. As in the Chinese study, we observed no association with ischaemic heart disease mortality. In accordance with both the American and the Chinese study, no association with cancer mortality was seen.

Fish consumption has often been associated with a healthy lifestyle [18, 22, 27], which is also indicated by our results; higher fish consumption corresponds to higher intake of fruit and vegetables and higher level of physical activity. However, all analyses were adjusted for possible confounders, but residual confounding may still be present and these results should therefore be interpreted with caution. Fish is highly recommended to prevent cardiovascular diseases [6, 49–51], and the joint FAO/WHO report also finds it convincing that maternal fish consumption contributes to optimal neurodevelopment in their offspring [49].

The present study does not support a protective effect of high fish consumption on death of all causes, or cause specific death. However, there is no reason to change the recommendations for fish intake. A higher consumption of fish may substitute for high intake of other presumably less healthy foods, e.g. processed meat; a product formerly found to give a higher risk of mortality [32].
